# A minimal T-cell immunogen designed to cover HIV-1 specificities associated with control is immunogenic in mice and breaks CTL immunodominance

**DOI:** 10.1186/1742-4690-9-S2-P305

**Published:** 2012-09-13

**Authors:** B Mothe, A Llano, M Rosati, S Perez-Alvarez, V Kulkarni, B Chowdhury, C Alicea, RK Beach, NY Sardesai, GN Pavlakis, BK Felber, C Brander

**Affiliations:** 1 IrsiCaixa AIDS Research Institute-HIVACAT, Barcelona, Spain; 2 NCI-Frederick, Frederick, MD, USA; 3 Inovio Biomedical Corp., Blue Bell, PA, USA; 4 IrsiCaixa AIDS Research Institute-HIVACAT, ICREA, Barcelona, Spain

## Background

Few T-cell immunogen vaccine designs exist that are based on large human immunogenicity data and which avoid inducing responses to mutable epitopes that may serve as immunodominant decoys. We have developed and tested a rationally designed T cell immunogen sequence which overcomes these limitations and which is currently undergoing pre-clinical testing.

## Methods

250 HIV-1 clade B infected individuals were screened for T cell responses to the entire HIV proteome. This yielded 26 regions in HIV-1 Gag, Pol, Vif and Nef proteins that were i) preferentially targeted by individuals with low viral loads, ii) more conserved and iii) elicited responses of higher functional avidity and broader cross-reactivity than responses to other, less-beneficial regions. The ‘beneficial’ segments were linked by triple alanines, translated into an expression-optimized nucleotide sequence and cloned into a CMV plasmid harboring a GM-CSF signal peptide. Immunogenicity was evaluated in C57BL/6 mice two weeks after a second DNA vaccination. Cellular immune responses were characterized using intracellular cytokine staining and IFN-γ ELISPOT using overlapping peptide pools covering the segments included in the T-cell immunogen.

## Results

Vaccination with 20 μg of DNA generated both CD4 and CD8 IFN-γ+ responses to the immunogen sequence. The T-cell immunogen elicited a more balanced, broad T cell response to all protein components (Gag, Pol, Vif and Nef) contained in the immunogen than immunizations using plasmids encoding for the entire Gag, Pol, Nef, Tat and Vif proteins, which induced a strong Gag dominance.

## Conclusion

Despite lower in vitro expression, the DNA vaccine was strongly immunogenic in C57BL/6 mice, induced broad CD4 and CD8 T cell responses and was able to break the immunodominance of responses to targets that do not emerge as particularly beneficial in large cohort screenings. Experiments in humanized BLT mice are currently ongoing to map induced responses in the context of different HLA genotypes.

